# Atomic-scale study clarifying the role of space-charge layers in a Li-ion-conducting solid electrolyte

**DOI:** 10.1038/s41467-023-37313-2

**Published:** 2023-03-24

**Authors:** Zhenqi Gu, Jiale Ma, Feng Zhu, Ting Liu, Kai Wang, Ce-Wen Nan, Zhenyu Li, Cheng Ma

**Affiliations:** 1 Hefei National Research Center for Physical Sciences at the Microscale, University of Science and Technology of China, Hefei, Anhui 230026 China; 2 CAS Key Laboratory of Materials for Energy Conversion, Department of Materials Science and Engineering, University of Science and Technology of China, Hefei, Anhui 230026 China; 3 Key Laboratory of Precision and Intelligent Chemistry, University of Science and Technology of China, Hefei, Anhui 230026 China; 4 School of Materials Science and Engineering, State Key Laboratory of New Ceramics and Fine Processing, Tsinghua University, Beijing, 100084 China; 5 Foshan (Southern China) Institute for New Materials, Foshan, Guangdong 528200 China; 6 National Synchrotron Radiation Laboratory, Hefei, Anhui 230026 China

**Keywords:** Batteries, Batteries, Batteries, Batteries

## Abstract

Space-charge layers are frequently believed responsible for the large resistance of different interfaces in all-solid-state Li batteries. However, such propositions are based on the presumed existence of a Li-deficient space-charge layer with insufficient charge carriers, instead of a comprehensive investigation on the atomic configuration and its ion transport behavior. Consequently, the real influence of space-charge layers remains elusive. Here, we clarify the role of space-charge layers in Li_0.33_La_0.56_TiO_3_, a prototype solid electrolyte with large grain-boundary resistance, through a combined experimental and computational study at the atomic scale. In contrast to previous speculations, we do not observe the Li-deficient space-charge layers commonly believed to result in large resistance. Instead, the actual space-charge layers are Li-excess; accommodating the additional Li^+^ at the 3c interstitials, such space-charge layers allow for rather efficient ion transport. With the space-charge layers excluded from the potential bottlenecks, we identify the Li-depleted grain-boundary cores as the major cause for the large grain-boundary resistance in Li_0.33_La_0.56_TiO_3_.

## Abstract (web summary)

Space-charge layers are believed to profoundly influence the interfaces in all-solid-state Li batteries. Here, the authors provide atomic scale insights into this phenomenon, and discover that its impact could be fundamentally different from commonly believed.

## Introduction

All-solid-state Li batteries are considered as an effective solution to the safety issues and limited energy density of commercial Li-ion batteries^1–3^, but their performances are often limited by the solid-solid interfaces with large resistance^4–6^. The concept of space-charge layers (SCLs) is frequently used to explain this phenomenon^5–7^. For example, when oxide cathodes and sulfide solid electrolytes contact each other, it is believed that their difference in electrochemical potential would drive a certain amount of Li^+^ from the sulfide to the oxide^4,5,8–11^. The resulting Li-deficient region, i.e., SCL, at the sulfide side does not possess enough charge carriers for efficient ion transport, and is thus considered the origin for the large interfacial resistance. Another example is the large grain-boundary resistance in many solid electrolytes^4,7,12,13^. The grain-boundary core of such materials is speculated to be positively charged, so Li-deficient SCLs that impedes ion transport would emerge nearby due to Coulomb repulsion. With SCLs broadly existent in different types of interfaces, precisely understanding their influence is indispensable for the rational performance optimization.

Nevertheless, presently one crucial factor is barely discussed in most (if not all) of the studies, and, as a result, the real influence of SCLs remains elusive. As mentioned above, the large interfacial resistance induced by SCLs is generally attributed to the existence of a Li-deficient region, whose low charge carrier concentration is believed to prevent decent ion transport^5,7,13^. However, in fact the low charge carrier concentration does not necessarily entail inefficient Li-ion migration, because the ion transport is dependent on many other factors too, such as the crystal structure, the Li distribution among different crystallographic sites, and the vacancy concentration. As a result, the structure with relatively low charge carrier concentration could still enable fast ion transport. For example, the perovskite-structured solid electrolyte Li_0.33_La_0.56_TiO_3_ is more conductive than the Li_0.5_La_0.5_TiO_3_ one with relatively high charge carrier concentration, because the latter does not possess sufficient A-site vacancies for Li ions to migrate through^14,15^. Another example is the Li_7_La_3_Zr_2_O_12_-based garnet solid electrolytes. By introducing Ta into the lattice, Li_7_La_3_Zr_2_O_12_ would become Li_6.5_La_3_Zr_1.5_Ta_0.5_O_12_ with lower Li-ion concentration, but in the meantime its crystal structure also changes from the less conductive tetragonal symmetry into the more conductive cubic one^16,17^. Consequently, the Li-deficient garnet Li_6.5_La_3_Zr_1.5_Ta_0.5_O_12_ turns out more conductive^17^. Clearly, by looking at the charge carrier concentration alone, one cannot tell whether the ion transport is efficient or not in SCLs; a proper understanding may only be reached through comprehensive investigation of the specific atomic configuration. In recent years, although more and more powerful techniques are demonstrated as effective tools for the characterization of SCLs^4,18^, their atomic configurations remain unexplored. Therefore, despite the large number of insightful computational studies in recent years^19–24^, the absence of experimental verification still prevents the conclusive, precise comprehension of the critical interfaces involving SCLs.

Here, based on direct observation of the atomic configuration, we clarify the role of SCLs in Li_0.33_La_0.56_TiO_3_ (LLTO), a prototype solid electrolyte plagued by the large grain-boundary resistance^12,19–21,25^, through a combined experimental and computational investigation. The study discloses a scenario that is completely different from the previous understanding. We do not observe the Li-deficient SCLs that have been broadly believed to exist; instead, we find that the grain-boundary SCLs are Li-excess. With the detailed atomic configuration within SCLs determined by aberration-corrected transmission electron microscopy, the ab initio molecular dynamics simulations unambiguously demonstrate that the SCLs, in sharp contrast to common expectations, actually exhibit satisfactory ion transport and cannot be the major cause for the large grain-boundary resistance. By clarifying the role of SCLs, the present study eventually identifies the grain-boundary cores as the major bottleneck for the sluggish ion transport.

## Results

### Li-excess or Li-deficient SCLs?

**Fig. 1:**
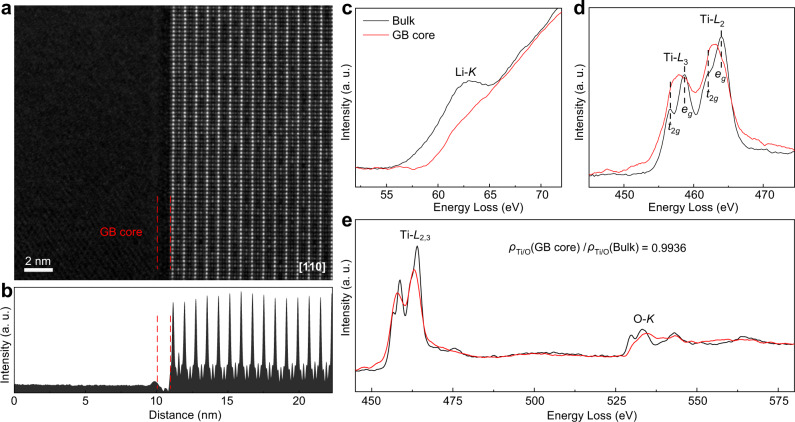
Probing the charge of the grain-boundary core. **a**, **b** HAADF-STEM image (**a**) and the corresponding accumulated intensity profile (**b**) of a grain-boundary (GB) core. The region indicated with red dashed lines is the grain-boundary core with darker contrast. **c**–**e** EELS data of Li-*K* (**c**), Ti-*L*_2,3_ (**d**), and O-*K* edges (**e**). To better illustrate the intensity of O-*K* with respect to that of Ti-*L*_2,3_, the range of energy loss in **e** is intentionally broadened to show the Ti-*L*_2,3_ edge in the same spectrum. *ρ*_Ti/O_(GB core) and *ρ*_Ti/O_(Bulk) represent the Ti-*L*_2,3_/O-*K* integrated intensity ratio of the grain-boundary core and that of the bulk, respectively.

In literature, the grain-boundary core of LLTO has been believed to be positively charged, so it would drive away the nearby Li ions, forming Li-deficient SCLs to impede ion transport^7,13,26^. Nevertheless, this scenario has never been verified by direct experimental observation; should the grain-boundary cores be negatively charged and create Li-excess SCLs instead, the influence on ion transport would be completely different. Consequently, our investigation begins with the experimental observation of the grain-boundary core and the nearby Li content fluctuation. The LLTO ceramic used for study was prepared by the common sintering method (details in Methods). According to X-ray diffraction (Supplementary Fig. 1) and electrochemical impedance spectroscopy measurement (Supplementary Fig. 2), its phase purity and ionic conductivity are both consistent with those reported in literature^7,27^. In order to determine whether the grain-boundary cores are positively or negatively charged, the atomic-resolution high-angle annular dark-field (HAADF) scanning transmission electron microscopy (STEM) imaging and the electron energy loss spectroscopy (EELS) measurement were conducted. Figure 1a shows the HAADF-STEM image of a typical grain boundary of LLTO. Consistent with previous observations^12,13^, the grain-boundary core appears darker than the bulk in this imaging mode (Fig. 1b). Given that the contrast of HAADF-STEM imaging is proportional to *Z*^1.7^ (*Z* is the atomic number)^28^ and La is the heaviest element in LLTO, the observed darkness entails that La is depleted at the grain-boundary core, in agreement with the previous reports too^12,13^. Beyond the La content, the variation of other elements was probed by EELS. The phenomena reported before for the grain-boundary core were all observed, i.e., the depleted Li (Fig. 1c), the slightly reduced Ti (indicated by the lower *L*_2_/*L*_3_ intensity ratio of the grain-boundary core in Fig. 1d), and the significantly changed environment of O (Fig. 1e). These results further confirm the earlier report that the grain-boundary core is essentially a TiO_*x*_ layer^12^. More importantly, the Ti-*L*_2,3_/O-*K* integrated intensity ratio suggests that the Ti/O ratio in the grain-boundary core is nearly the same as that in the bulk (Fig. 1e). That is, the value of *x* in the TiO_*x*_ layer constituting the grain-boundary core should be close to 3. Considering that the valence of Ti in the grain-boundary core is slightly below that in the bulk (4+) as mentioned above, the grain-boundary core should be negatively charged.

**Fig. 2:**
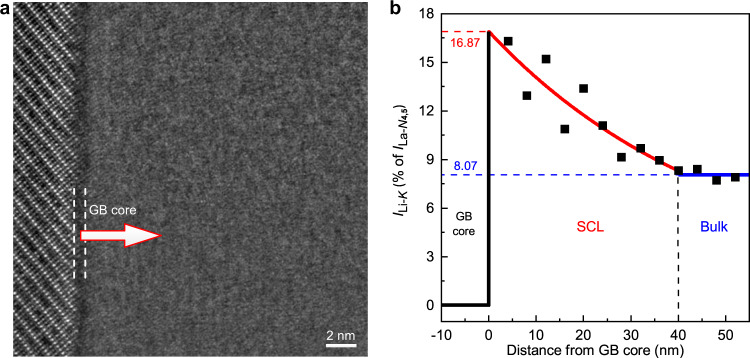
Li distribution in the vicinity of the grain-boundary core. **a** HAADF-STEM image of the region selected for the EELS line-scan experiment. GB stands for “grain boundary”. The arrow indicates the direction where the EELS line scan was conducted. **b** Variation of the normalized Li-*K* intensity, *I*_Li-*K*_, with the distance from the grain-boundary (GB) core. SCL represents “space-charge layer”. The solid squares are the normalized Li-*K* intensity acquired from the EELS line-scan experiment. The black line is the normalized Li-*K* intensity within the GB core. The red curve is the exponential fit conducted to the points between 0 and 40 nm. The blue line denotes the normalized Li-*K* intensity in bulk, i.e., 8.07%.

Consistent with the negative charge observed for the grain-boundary core, the neighboring regions were found enriched with Li. Figure 2 shows the representative EELS results in the vicinity of a grain boundary, where the spectra were collected every 4 nm along the line indicated by the arrow in Fig. 2a. It should be noted that the EELS intensity reflects the content, rather than the concentration, of the corresponding element in a given area. Therefore, it will be affected by the specimen thickness, and cannot straightforwardly indicate the composition variation. In order to avoid this distraction, the discussion on the Li concentration below would not focus on the as-measured Li-*K* intensity, but on the one normalized to the integrated intensity of the La-*N*_4,5_ edge in the same spectrum. Such a normalized Li-*K* intensity reflects the Li content with respect to that of La, so it is independent of the specimen thickness; in the meanwhile, since the La concentration barely fluctuates near the grain-boundary core (as indicated by the constant image intensity of the La columns, i.e., the bright spots, in Fig. 1a), the variation of the normalized Li-*K* intensity mentioned above can still truthfully reflect the change of the local Li concentration. In Fig. 2b, the normalized Li-*K* intensity is presented as the percentage of the corresponding La-*N*_4,5_ intensity and is plotted against the distance from the grain-boundary core. Compared with the bulk (normalized Li-*K* intensity 8.07%), the vicinity of the grain-boundary core is clearly Li-rich, where the maximum normalized Li-*K* intensity exceeds 16%. The Li concentration is highest at the location closest to the grain-boundary core, and gradually decreases with increasing distance; the variation tendency largely follows an exponential curve, consistent with that described by the SCL theory^29^. When the distance from the grain-boundary core is larger than 40 nm, the normalized Li-*K* intensity eventually stabilizes at the bulk level, i.e., 8.07%.

The results above unambiguously indicate that the actual SCL in LLTO is fundamentally different from the scenario proposed in literature. While the existing studies believe that the grain-boundary cores are positively charged and make the neighboring regions Li-deficient^7^, the experimental observation here suggests they are in fact negatively charged and result in Li enrichment nearby. While in literature the SCLs of LLTO were estimated to be as narrow as 5.5 nm^7^, the actual SCLs were found to exhibit a much larger width of around 40 nm (Fig. 2b). With both the charge and Li distribution demonstrated to be different from previous speculations, the influence of SCLs on ion transport can never be the same, and a more in-depth investigation seems necessary. To this end, the atomic configuration of the SCLs, especially the distribution of excess Li within the lattice, must be thoroughly studied.

### Atomic configuration of the SCLs

Although the overall atomic framework of the SCL region remains perovskite, it needs to accommodate a lot more Li ions. The EELS results in Fig. 2b suggest that the highest Li concentration in the SCL is nearly twice that in the bulk (normalized Li-*K* intensity 16.87 vs 8.07%). Considering that the bulk exhibits 0.33 Li per unit cell (unless otherwise specified, the “unit cell” in this work refers to the prototype perovskite unit cell with one ABX_3_ formula unit), the maximum number of Li per unit cell in the SCL region should be around 0.66; that is, the Li concentration in SCLs varies between 0.33 and 0.66 Li per unit cell. Nevertheless, even if all the A-site vacancies in a Li_0.33_La_0.56_TiO_3_ unit cell are occupied, the number of Li may only reach 0.44. For the region containing more Li, their location in the unit cell needs to be properly determined.

The lithiation experiment of the perovskite-structured solid electrolyte may shed light on the investigation of this issue^30^; in this experiment, Li_0.37_La_0.50_TiO_2.94_, a perovskite with similar composition with the Li_0.33_La_0.56_TiO_3_ studied here, was cycled as a cathode in an electrochemical cell. When the discharge capacity of Li_0.37_La_0.50_TiO_2.94_ reaches the value corresponding to the full occupation of its A-site vacancies, it was found that the lithiation can further proceed at a slightly different voltage. The Li inserted at this stage was speculated to occupy the “3c site” of the cubic perovskite structure, which lies between two La-poor A-sites, as schematically illustrated in Supplementary Fig. 3. This site can accommodate up to 3 Li per unit cell, whereas the number of Li that have to reside at interstitials is much smaller; since each unit cell in the SCLs contains no more than 0.66 Li and 0.44 of them can be hosted by A-sites, the interstitial Li in the SCLs would not exceed 0.22 per unit cell. Therefore, a hypothesis can be raised: the Li distribution in the unit cell of SCLs could be similar to that in the lithiated Li_0.37_La_0.50_TiO_2.94_. That is, the regions with Li concentration below 0.44 per unit cell would simply host Li at the A-sites, and those with Li concentration above this value would have the additional Li occupy the 3c interstitial sites.

**Fig. 3:**
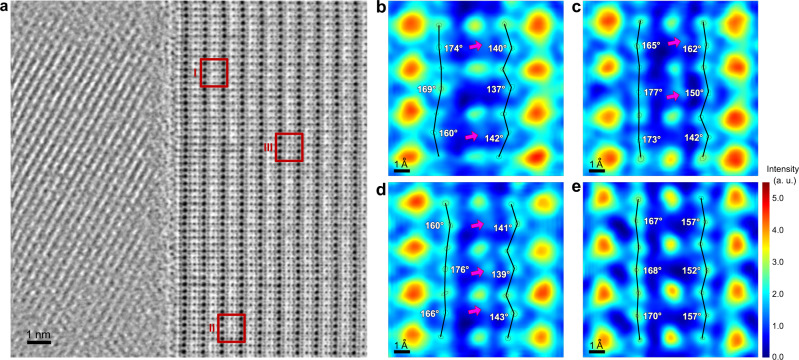
Atomic configuration in the SCL. **a** ABF-STEM image of the vicinity of a grain-boundary core. The grain at the right was observed along <110 > _p_. **b**–**e** Enlarged ABF-STEM images of regions I (**b**), II (**c**), and III (**d**) in **a**, along with one taken from the bulk (**e**). The magnifications of these enlarged images are the same. For clarity, the images are presented in false colors. The Ti-O bonds are represented by the black lines. The interstitial Li are arrowed in red. The Ti-O-Ti angels were determined by CalAtom.

In order to experimentally verify the hypothesis raised above, the adopted microscopy technique not only needs to reach atomic resolution, but also must be able to visualize Li. The HAADF-STEM imaging used in Figs. 1 and 2 is almost blind to elements with overly small atomic number because its intensity is proportional to *Z*^1.7^ and decays too fast with the decrease of the atomic number^28,31^. Therefore, annular bright-field (ABF) STEM imaging was used instead; its image intensity is dependent on *Z*^1/3^, and can still generate observable contrast for light elements such as Li^28,31^. In addition to the proper imaging technique, the crystallographic orientation for observation also needs to be selected wisely. As schematically illustrated in Supplementary Fig. 4a, if the observation is conducted along <100 > _p_ (the subscript p indicates that the index follows the cubic prototype perovskite unit cell), the interstitial 3c site would be overlapped with O. In this way, no matter whether this site contains Li, the corresponding spot in the image would always display non-negligible contrast due to the co-existence of O in the same atomic column. Such a distraction may only be avoided by performing the observation along a direction where the 3c sites do not overlap with any other atoms. Fortunately, the <110 > _p_ axis meets this requirement. As shown in Supplementary Fig. 4b, the unit cell viewed along <110 > _p_ displays atomic columns formed by the 3c sites only. If any contrast was observed there, there must be Li lying at the corresponding 3c site. With the imaging mode and orientation both properly selected, the atomic-resolution observation was conducted. The ABF-STEM image of the vicinity of a grain-boundary core is shown in Fig. 3a, where the grain at the right side is oriented along the <110 > _p_ axis mentioned above. Since La is the heaviest element in LLTO and the image contrast increases with the atomic number^28,31,32^, the arrays of darkest, largest spots in this <110 > _p_-oriented grain correspond to the La-rich layer, and the arrays of lighter spots located halfway between two La-rich layers are the La-poor ones. By comparing the ABF-STEM image in Fig. 3a and the atomic model in Supplementary Fig. 4b, it can be found that the 3c interstitials should lie between two La-poor spots. A close examination of such regions in the ABF-STEM image suggests that most unit cells near the grain-boundary cores indeed possess Li in their 3c sites, as exemplified by regions I, II, and III in Fig. 3a (their enlarged images are displayed in Supplementary Fig. 5a–c, respectively). As pointed out by the red arrows, Li ions were observed in the 3c interstitial sites of virtually all the unit cells. In comparison, the same atomic sites in the bulk region were never observed to exhibit Li (Supplementary Fig. 5d and Supplementary Fig. 6). This observation confirms the hypothesis raised above: the excess Li that cannot be hosted by the A-sites in the SCLs would be accommodated by the 3c interstitials. Furthermore, the microscopy observation discloses several interesting behaviors for these interstitial Li; in order to visualize them more clearly, the images in Supplementary Fig. 5a−d are presented in false color and displayed in Fig. 3b−e, respectively. First of all, it appears that the actual locations of the interstitial Li are slightly different from that in the standard atomic model. The observed Li spots in Fig. 3b−d are in fact shifted slightly to the right with respect to the standard 3c interstitial location indicated in Supplementary Fig. 4b. This might be caused by the size difference between the 3c interstitial and Li^+^; the former is in fact the largest interstitial in the perovskite structure^30^, and might be too large for the latter to fit precisely in. Secondly, the locations of interstitial Li in different unit cells do not seem exactly the same. In some images, the interstitial region appears to contain more than one spots, as exemplified by the central area in Fig. 3c. Since the Coulomb repulsion forbids one 3c interstitial to host two Li ions simultaneously, the additional spot should arise from the other unit cell along the observation direction, entailing that the exact positions of interstitial Li are not always the same in the SCL. Last but not least, the presence of Li would distort the nearby atomic configuration. Using the CalAtom software^33^, we have calculated the variation of the Ti-O-Ti angles from 133 unit cells in the SCL region and the bulk region, respectively, and a few angles are denoted in Fig. 3b−e. In the bulk, the ranges for the Ti-O-Ti angles in the two Ti-O layers near the interstitial Li are 157−180° and 149−179°, respectively. In comparison, the values in the SCL region become 130−180° and 121−173°, respectively. This difference further supports the existence of Li at 3c interstitials; after all, with the originally empty atomic sites occupied by Li, the change of the nearby atomic configuration is inevitable. In addition to the interstitial Li, the grain-boundary core that is structurally different from the bulk might also induce strains in the unit cells of the SCL. In order to investigate this effect, we examined the unit cells with no interstitial Li in Fig. 3a, and analyzed the evolution of their volumes with the distance from the grain-boundary core. By conducting the multiple-ellipse fitting using the CalAtom software^33^, the positions of the atomic columns (and thus the lattice parameters) can be accurately determined for all the unit cells. The fitting result is displayed in Supplementary Fig. 7, with each column of unit cells numbered. Since the grain-boundary core here happens to be parallel with the (001) plane of the grain at the right, the unit cells within any individual column in Supplementary Fig. 7 are at the same distance from the grain-boundary core. For each column, the number of unit cells without interstitial Li and the average volume of such unit cells are summarized in Supplementary Table 1. The results do not disclose any correlation between the volumes of these unit cells and their distances from the grain-boundary core; instead, the former only show limited fluctuation in a rather random manner. Therefore, the difference between the atomic configuration of the grain-boundary core and that of the SCL does not impose non-negligible strain in the latter. In fact, the grain-boundary core with such a different atomic configuration from that of the bulk has been proposed to alleviate the strain in the two adjacent grains instead^12^. Based on these results, the strain in the SCLs should arise mostly (if not exclusively) from the presence of excessive Li. Finally, it should be emphasized that, although the discussion above are centered on one grain boundary, similar phenomena have been observed for all the grain boundaries we examined (20−30 in total); a few more examples are displayed in Supplementary Figs. 8−11. According to such repeated observation, the grain boundaries may virtually show any orientation, without noticeable preference, but the SCLs were always found to exhibit interstitial Li near the 3c sites. Although the specific locations of the interstitial Li and the extent of the associated lattice distortion vary slightly among the SCLs of different grain boundaries, no fundamental distinction was identified. The observations above suggest that the SCLs are not only widely existent but also show very different atomic configurations from those of the bulk. Straightforwardly determining the net contribution of these structural features to the ion transport seems challenging. Therefore, a computational investigation is conducted.

### Li^+^ migration in the SCLs

**Fig. 4:**
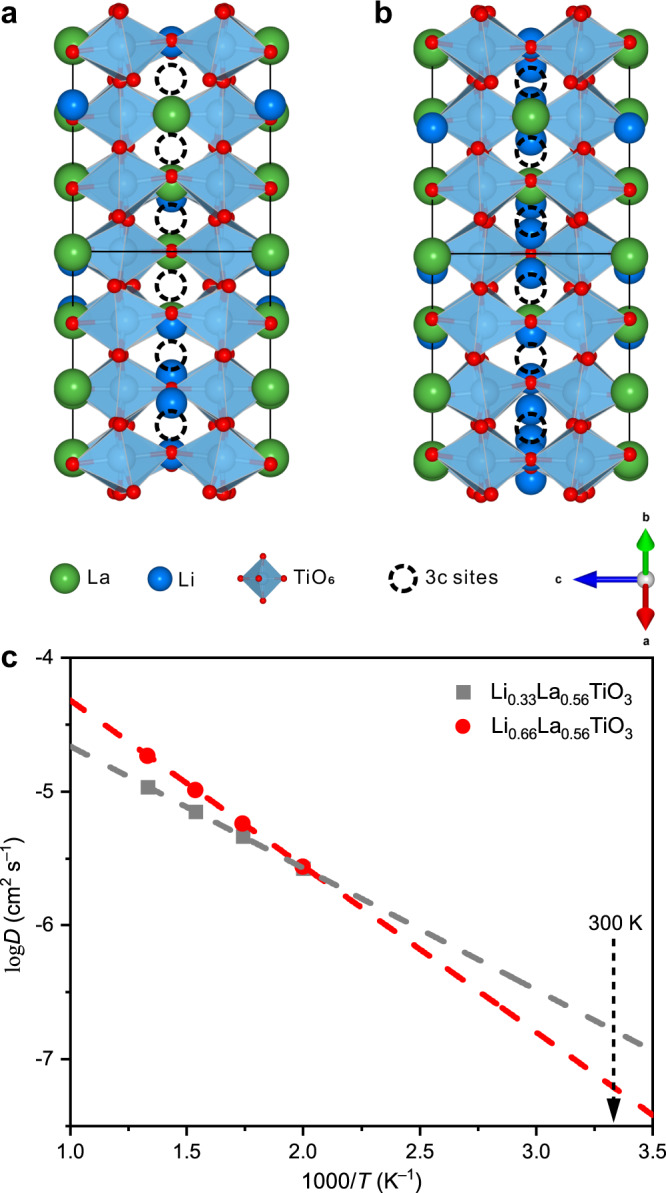
AIMD simulations of the Li-ion transport. **a**, **b** Optimized models of Li_0.33_La_0.56_TiO_3_ (**a**) and Li_0.66_La_0.56_TiO_3_ (**b**) viewed along <110>_p_. The 3c interstitial sites are marked by dashed circles. **c** Arrhenius plots of the calculated diffusion coefficients *D* for Li_0.33_La_0.56_TiO_3_ and Li_0.66_La_0.56_TiO_3_. The diffusion coefficients at 500, 575, 650, and 750 K were obtained directly from AIMD simulations, and those at 300 K were inferred by fitting the high-temperature data using the Arrhenius relationship.

In order to probe the ion transport behavior within the SCLs through first-principles calculations, we constructed an atomic model for Li_0.66_La_0.56_TiO_3_, which represents the region showing the highest Li concentration in the SCL; for comparison, the atomic model for the bulk region, i.e., Li_0.33_La_0.56_TiO_3_, was also constructed. The structural models for computation here do not include the grain-boundary cores but contain only the unit cells within the SCLs, for two reasons. First of all, the specific atomic configuration of the grain-boundary core depends on how the two neighboring grains are aligned with respect to each other, but the grains in the actual material are randomly oriented, resulting in considerable variation among the structures of different grain-boundary cores. Consequently, it is unlikely (if not impossible) to find a structural model that can well represent all or at least most of the grain-boundary cores. Secondly, it was found that the specific atomic configurations of the grain-boundary cores would not fundamentally change the structures of the nearby SCLs. In the present study, we have examined 20−30 grain boundaries in total. Although the orientations of the grains separated by these grain boundaries (and thus the atomic configurations of the grain-boundary cores) vary significantly, the SCLs always exhibit the structure similar to that in Fig. 3, where interstitial Li are present near the 3c sites; a few examples are shown in Supplementary Figs. 8−11. Since the specific atomic configurations of the grain-boundary cores can neither be properly represented by any structural model nor induce non-negligible change to the SCLs, i.e., the regions of interest in the present study, it appears more reasonable not to take them into account during simulation. Instead, the computational investigation would focus on the SCLs (represented by the aforementioned Li_0.66_La_0.56_TiO_3_ model) and the bulk structure for comparison (represented by the aforementioned Li_0.33_La_0.56_TiO_3_ model). Starting with about 10 to 20 randomly generated initial structures, the geometry of each structure was optimized with the density functional theory (DFT) calculation. The optimized structures agree well with the electron microscopy observation. The Li_0.33_La_0.56_TiO_3_ model accommodates Li with its A-sites (Fig. 4a), consistent with the bulk structure. As for the Li_0.66_La_0.56_TiO_3_ model representing the SCL structure, its large amount of Li cannot all be hosted by the A-sites, and the additional ones reside near the 3c interstitial sties, with the specific location varying among different unit cells (Fig. 4b). This is in good agreement with the repeated electron microscopy observation of the 20−30 SCL regions examined in the present study (Fig. 3 and Supplementary Figs. 8−11).

With the optimized geometry described above as the initial structure, ab initio molecular dynamics (AIMD) simulations were conducted to investigate the Li-ion transport behavior. The simulation does not take into account the electric fields, because their influence will be canceled by that of the Li-ion concentration gradient in the SCL. More specifically, while the negatively charged grain-boundary core studied here is attracting Li ions, the local accumulation of Li ions caused by this effect is forming a negative concentration gradient (Fig. 2b) that pushes Li ions away from the grain-boundary core in the meantime. At equilibrium, the influence of the electric fields would be balanced by that of the concentration gradient^34,35^. Consequently, neither of them can significantly affect ion transport. Since the direct computation of Li-ion diffusion at 300 K requires an unaffordable time to converge, the AIMD simulations were performed at an intermediate temperature range of 500−750 K instead, and the room-temperature diffusion behavior was inferred by fitting the high-temperature data points using the Arrhenius relationship. The evolution of the mean square displacement (MSD) for Li_0.33_La_0.56_TiO_3_ and Li_0.66_La_0.56_TiO_3_ at different temperatures are displayed in Supplementary Fig. 12, and the Li-ion diffusion coefficients were calculated accordingly from these data (details in Methods). It should be noted that, due to the layered nature of the tetragonal perovskite structure studied here, the diffusion during the AIMD simulation occurred exclusively within the (001) plane. Therefore, although the equation used for calculating the diffusion coefficients is three-dimensional, the values obtained from the simulation are in fact contributed by the two-dimensional diffusion within (001). For the same reason, the diffusion coefficients parallel and perpendicular to the grain boundary are dependent on how the grain boundary is oriented with respect to (001), so the former is not necessarily always larger or smaller than the latter in different grains. The Li-ion diffusion coefficients calculated from the MSDs in Supplementary Fig. 12 agree well with the Arrhenius relationship for both Li_0.33_La_0.56_TiO_3_ and Li_0.66_La_0.56_TiO_3_ (Fig. 4c), so extrapolating such data should provide an acceptably accurate estimation of the diffusivities at low temperatures. Based on the diffusion coefficients at 300 K, the room-temperature ionic conductivities were calculated through the Nernst-Einstein equation (details in Methods). Following this procedure, Li_0.33_La_0.56_TiO_3_ was found to show an ionic conductivity of 1.906 × 10^–3^ S cm^–1^ at 300 K, consistent with the experimentally measured bulk conductivity (8.95 × 10^–4^ S cm^–1^, Supplementary Fig. 2). In comparison, the ionic conductivity of Li_0.66_La_0.56_TiO_3_ at 300 K was 7.166 × 10^–4^ S cm^–1^; for the regions with lower Li concentration, the conductivity should supposedly be closer to that of the bulk. That is, the ionic conductivity of the SCL region at 300 K should lie between 7.166 × 10^–4^ and 1.906 × 10^–3^ S cm^–1^, which is not so different from that of the bulk (1.906 × 10^–3^ S cm^–1^).

The computational results presented above are supported by experiments. In order to measure the ion transport efficiency in the structures with excessive Li, we synthesized two more materials: Li_0.44_La_0.56_Ti_0.88_Al_0.12_O_3_ and Li_0.55_La_0.56_Ti_0.77_Al_0.23_O_3_. They are both isostructural with Li_0.33_La_0.56_TiO_3_ (Supplementary Fig. 13a), but possess excessive Li, resembling the SCLs studied above; although the B-sites of these two Li-excess materials host a certain amount of Al^3+^, instead of being fully occupied by Ti^4+^ like those in Li_0.33_La_0.56_TiO_3_, the small molar fraction of Al^3+^ and the similarity between the ionic radii of these two cations^36^ should ensure that the Li-ion migration efficiency within their grain bulk does not deviate considerably from that in the Li-excess SCLs studied above. Based upon this fact, the ion transport behavior was studied by electrochemical impedance spectroscopy. The Nyquist plots of the two Li-excess materials both exhibit clearly distinguishable grain-boundary and bulk semicircles, just like that of the stoichiometric Li_0.33_La_0.56_TiO_3_ (Supplementary Fig. 13b). From such data, the bulk conductivities at 25 °C were found to barely vary with the Li content (Supplementary Fig. 13c), consistent with the computational results presented above. In particular, if the B-sites of Li_0.44_La_0.56_Ti_0.88_Al_0.12_O_3_ and Li_0.55_La_0.56_Ti_0.77_Al_0.23_O_3_ do not have to host a certain amount of Al^3+^ to maintain the charge balance, but may be fully occupied by the slightly larger Ti^4+^ like those in the SCLs of Li_0.33_La_0.56_TiO_3_, their unit cells could be larger, leading to ionic conductivities higher than those shown in Supplementary Fig. 13c and thus even closer to that of Li_0.33_La_0.56_TiO_3_. Generally speaking, these experimental results corroborate the computational ones presented above: the ion transport within the SCLs is almost as efficient as that in the bulk. Such a phenomenon may be comprehended by comparing the highest allowable Li content in the unit cell and the actual ones. The Li_0.33_La_0.56_TiO_3_ material can host up to 3.44 Li per perovskite unit cell with its A-sites and 3c interstitials. Compared with this value, the Li contents in the SCLs (0.33−0.66 per perovskite unit cell) are in fact rather small, and the variation is also very limited. Therefore, the ion transport efficiency is unlikely to undergo considerable fluctuation.

The observations above suggest that the SCLs alone cannot possibly account for the large grain-boundary resistance observed in the Nyquist plot (Supplementary Fig. 2). Before elaborating on this point, it should first be pointed out that the “grain-boundary conductivity” determined from the “grain-boundary semicircle” in the Nyquist plot is in fact a fundamentally different physical quantity from the ionic conductivity discussed in the calculations above. The grain-boundary semicircle in the Nyquist plot reflects the contribution of all the grain boundaries to the total resistance; it is influenced not only by the ion transport efficiency of each grain boundary, but also by the population of the grain boundaries. In contrast, the conductivity discussed in the AIMD simulations above describes the ion transport within a given structure only, and is not affected by the amount of the corresponding components. For clarification, this conductivity is referred to as the “intrinsic conductivity” below, while that determined from the semicircle in the Nyquist plot would be referred to as the “Nyquist conductivity”. If the “Nyquist conductivity” for grain boundaries (5.59 × 10^−5^ S cm^–1^, Supplementary Fig. 2) arose exclusively from the SCLs, i.e., a component with negligibly small dimension compared to that of the grains (40 nm vs. 2−4 μm), the “intrinsic conductivity” of SCLs would have to be even lower than 5.59 × 10^−5^ S cm^–1^, possibly by orders of magnitude. However, the AIMD simulations above suggest just the opposite; the “intrinsic conductivity” of SCL is no lower than 7.166 × 10^–4^ S cm^–1^, so they cannot be the major cause for such large grain-boundary resistance. As a matter of fact, considering that the ion transport in SCLs is almost as fast as that in the bulk and the volume fraction of SCLs is also rather low, their influence on the total ionic conductivity should be very limited^12^. This is in sharp contrast to the previously proposed scenario, where the large grain-boundary resistance of LLTO was believed to arise mainly from the SCLs^7^.

### Actual bottleneck for the grain-boundary ion transport

Although the present study was meant to focus on the role of SCLs, the clarification of this issue enables the identification of the actual cause for the large grain-boundary resistance as well. In literature, the possible origins for the sluggish grain-boundary ion transport are not limited to the SCLs. Instead, the Li-depleted grain-boundary cores like those shown in Figs. 1 and 2 were also proposed as a potential contributor; their locally reconstructed atomic configuration was believed to forbid the existence (and thus the transport) of Li ions^12^. Prior to the present study, both the SCLs and the grain-boundary cores could possibly be the bottlenecks, and it is difficult to tell whether the large resistance is contributed by both, or mostly by one of them. Consequently, the ion transport mechanism remains elusive. Nevertheless, now that the SCLs have been demonstrated to barely impede the overall Li-ion transport, the grain-boundary cores become the only possible bottleneck. This scenario is also supported by the observations in the present study. As shown in Fig. 2b, the grain-boundary core barely contains any Li, while the SCL regions closest to the grain-boundary core is highly Li-rich. Such a large, sharp concentration gradient between the SCL and the grain-boundary core would tend to push Li ions into the latter. Additionally, the negative charge at the grain-boundary core would result in the same effect on the nearby positively charged Li ions too. Regardless, even under the strong driving forces from both the concentration gradient and the Coulomb interaction, Li ions still cannot enter the grain-boundary core; according to our observation (Fig. 1) and that reported previously^12^, most grain-boundary cores are Li-depleted. Such a strong resistance to the presence of Li ions at the grain-boundary cores would inevitably create a great hindrance to ion transport. In particular, with the SCLs excluded from the possible bottlenecks, the Li-depleted grain-boundary cores can now be identified as the major origin for the large resistance, rather than one of the possible contributing factors. Beyond the LLTO material studied here, it should be pointed out that the ion transport of other ceramic solid electrolytes could be severely impeded by the grain-boundary cores too. In order to exhibit meaningful ionic conductivity, ceramic solid electrolytes such as LLTO, Li_7_La_3_Zr_2_O_12_, and Li_1+*x*_Al_*x*_Ti_2−*x*_(PO_4_)_3_ need to be densified through sintering at elevated temperatures^37^. If a ceramic contains volatile elements like Li, K, and Bi, it would lose these elements at the high sintering temperatures, preferentially through grain boundaries^38–40^. As a result, the grain-boundary cores in most ceramic solid electrolytes should be Li-poor like those in LLTO, and would thereby degrade the Li^+^ migration efficiency through a similar mechanism. With the capability of impeding ion transport in different ceramic solid electrolytes, such grain-boundary cores deserve more in-depth investigation in future studies.

As for the possible improvement strategies, the nature of the Li-depleted grain-boundary core is in fact indicative of several potential approaches already. According to Chi et al.^12^, the local structural reconstruction that forbids the existence or transport of Li ions in the grain-boundary core happens because of the need to reconcile the local distinction between the atomic configurations of the two differently oriented grains. Therefore, if the grains can be separated by a sufficiently Li-ion conductive intergranular phase, instead of being in direct contact with each other, the aforementioned grain-boundary structural reconstruction can be avoided. In fact, even if the amorphous silica, a compound with relatively poor Li-ion transport efficiency, is present between the adjacent grains, the grain-boundary resistance can still be reduced^41^. Therefore, introducing intergranular phases should be an effective approach. Additionally, it has also been observed that appropriately oriented grains and grain-boundary cores may also prevent the undesired structural reconstruction mentioned above^12^. For example, if the grain-boundary core happens to be simultaneously parallel with the (011) plane of one grain and the (001) plane of the other, the two neighboring grains may match semi-coherently with each other and thus eliminate the need for severe local reconstruction^12^. If certain synthesis or processing approaches can be developed to effectively control the orientations of the grain-boundary cores and the neighboring grains, the ion transport efficiency might also be greatly improved.

Although the observation here was conducted on only one material, Li_0.33_La_0.56_TiO_3_, it points out the necessity and urgency of experimentally examining the SCLs of different interfaces at the atomic scale, and thus would benefit the research of solid electrolytes in general. For decades, the SCLs of Li-ion-conducting solid electrolytes have been comprehended mostly (if not exclusively) from the perspective of Li-ion concentration, and the present interpretation of the behaviors of many critical interfaces are also based upon such understanding^4,5,7^. Regardless, by examining the specific atomic configuration, it was immediately found that the role of SCLs in Li_0.33_La_0.56_TiO_3_ is opposite to that commonly believed. Considering that the SCLs in many other technologically important interfaces (such as those between the 4 V-class cathodes and sulfide solid electrolytes) have also been comprehended without considering the atomic structure^4,6,11,42–44^, the current understanding could deviate from the actual mechanism as well. In particular, the electrode-electrolyte interfaces, unlike grain boundaries, involve two materials (the cathode and the solid electrolyte), and the Li-ion concentration variation would most likely lead to different consequences to the materials with different crystal structures too. In order to reach a comprehensive understanding on these complicated interfaces, the mechanisms have to be studied on a case-by-case basis. Consequently, the atomic-scale investigation similar to that presented here needs to be conducted to as many interfaces as possible.

## Discussion

In summary, the present study investigates the influence of SCLs on the ion transport of LLTO from a largely ignored but crucially important perspective: the atomic configuration. According to the electron microscopy observations, the grain-boundary cores are negatively charged and create Li-rich SCLs nearby, which is just the opposite of the previous speculation^7,13^. The overall atomic framework in the SCL region remains perovskite, but the charge carrier concentration is much higher than that in the bulk. As a result, the Li in SCLs cannot be hosted by the A-sites alone. After A-sites are fully occupied, the additional Li reside near the 3c interstitials. According to the AIMD simulations based on the observed structure, the room-temperature ionic conductivity of the SCL region was found rather close to that of the bulk. Taking into account the small volume of SCLs with respect to that of the grains too (40 nm vs. 2−4 μm), the influence of SCLs on the overall ionic conductivity should be highly limited, and they cannot possibly be the major cause for the large grain-boundary resistance. Instead, the actual bottleneck for the sluggish grain-boundary ion transport should be the Li-depleted grain-boundary core. These results suggest that the comprehension of ion transport in SCLs must not focus on the Li concentration alone. Instead, properly studying the atomic configuration is indispensable. Beyond the grain boundaries, the present understanding of many other types of interfaces in all-solid-state Li batteries is also based on SCLs, so similar studies seem necessary for those subjects too.

## Methods

### Materials and macroscopic characterizations

The Li_0.33_La_0.56_TiO_3_ ceramics used in the present study were prepared using the same method as that in our previous work^27^. Specifically, the synthesis began with dissolving stoichiometric amounts of LiNO_3_ and La(NO_3_)_3_·6H_2_O in ethylene glycol monomenthyl ether. The solution was then mixed with tetrabutyl titanate and acetylacetone, and dried at 70 °C to form the gel. After the gel was calcined at 900 °C for 6 h, the resulting powder was ball milled for 12 h, pressed into a pellet, sintered at 1350 °C for 6 h, and finally annealed at 800 °C for three days. During these heat treatments, the pellets were buried in powders with the same composition to compensate for the Li loss. The stoichiometry of the acquired ceramic was confirmed by the inductively coupled plasma spectroscopy. The crystal structure and the ionic conductivity were determined by X-ray diffraction and electrochemical impedance spectroscopy, respectively.

### Electron microscopy

TEM specimens were prepared by mechanically thinning and Ar-ion milling. The ion milling was performed at 4 kV and 1.8 mA until perforation, and then a weaker beam of 0.8 kV and 0.8 mA was applied to remove the amorphous layer at the surface. The ion-milled specimens were stored in a 10^−5^ torr vacuum until being observed by electron microscopy. The STEM and EELS studies were conducted on an aberration-corrected FEI Titan Themis TEM/STEM equipped with a Gatan Image Filter Quantum-965. The observation was conducted at 200 kV. The HAADF and ABF images shown here were Fourier-filtered to minimize the contrast noise without the introduction of any artifact that may alter the conclusions. The positions of atomic columns in the ABF-STEM image were determined by the multiple-ellipse fitting method using the CalAtom software^33^. The EELS data were acquired in the STEM mode with a 5 mm aperture and an energy dispersion of 0.1 eV per channel.

### First-principles computation

All the ab initio molecular dynamics simulations were performed with the Vienna ab initio simulation package (VASP)^45,46^. The generalized gradient approximation (GGA) parametrized by Perdew, Burke, and Ernzerhof (PBE) was adopted to the exchange−correlation functional^47^. The projector-augmented wave (PAW) method was applied to describe the core-valence interaction^48^. The 1*s*^2^2*s*^1^, 5*s*^2^5*p*^6^5*d*^1^6*s*^2^, 3*p*^6^3*d*^2^4*s*^2^ and 3*s*^2^3*p*^4^ electrons were treated as the valence electrons for Li, La, Ti, and O, respectively. The energy cutoff was set to be 520 eV, and a 3 × 3 × 5 Monkhorst–Pack k-point grid was used to sample the Brillouin zone for the geometry optimization^49^. The convergence criteria for the electronic minimization and the geometry optimization were set to be 1 × 10^–5^ eV and 0.01 eV/Å, respectively. All the simulations were performed in the NVT ensemble with only the gamma point in the Brillouin zone sampling. The temperature was maintained by a Nose-Hoover thermostat^50^ with an integration time-step of 1 fs. Following a 10 ps equilibrium run, a 100 ps production run was generated for the statistics of Li-ion diffusion analysis. A 3 × 3 × 2 supercell was built for the simulation of each structural model. The size of the simulation unit was 11.636 × 11.636 × 7.871 Å^3^. Since the SCLs adjacent to the grain boundary are positively charged, four electrons were removed in the ab initio molecular dynamics simulations of the Li_0.66_La_0.56_TiO_3_ system. The diffusion coefficient *D* of Li was calculated by counting the mean square displacement as a function of the observation time:

1 $$D=\frac{1}{2d}\mathop{{{{{\mathrm{lim}}}}}}\limits_{t\to {{\infty }}}\frac{\left\langle {\left[\vec{{{{{{\bf{r}}}}}}}({t}_{{{{{{\rm{O}}}}}}}+t)-\vec{{{{{{\bf{r}}}}}}}({t}_{{{{{{\rm{O}}}}}}})\right]}^{2}\right\rangle }{t}$$

where *d*, $\vec{{{{{{\bf{r}}}}}}}({t}_{{{{{{\rm{O}}}}}}})$, and $\vec{{{{{{\bf{r}}}}}}}({t}_{{{{{{\rm{O}}}}}}}+t)$ are the dimensionality of the system, the position of Li at the time origin *t*_O_, and that at an observation time *t* after *t*_O_, respectively. The angled bracket indicates an ensemble average was taken over all Li atoms and all time origins. Any time in the production run can be considered as the time origin in the equation above. The ionic conductivity was calculated based on the Nernst−Einstein equation^51^:

2 $$\varLambda=\frac{\rho {{{{{{\rm{F}}}}}}}^{2}{z}^{2}}{{{{{{\rm{R}}}}}}T}D$$

where *ρ*, F, *z*, R, and *T* are the molar density of the charge carriers, the Faraday constant, the charge of Li^+^, the gas constant, and the temperature, respectively.

## Supplementary information

Supplementary Information

## Data Availability

The data that support the findings of this study are available within the article (and its Supplementary Information files) and from the corresponding authors upon reasonable request.
