# Supplementary material for: Atomic-scale study clarifying the role of space-charge layers in a Li-ion-conducting solid electrolyte
Source: Nat Commun. 2023 Mar 24;14:1632. doi: 10.1038/s41467-023-37313-2 (PMC10039002; doi:10.1038/s41467-023-37313-2)
Supplement: Supplementary file 1 — Supplementary Information [file 41467_2023_37313_MOESM1_ESM.pdf]

Supplementary Information

Atomic-scale study clarifying the role of space-charge layers in a Li-ion-conducting solid electrolyte

Zhenqi Gu[1,2†], Jiale Ma[3†], Feng Zhu[1,2†], Ting Liu[4,5], Kai Wang[1,2], Ce-Wen Nan[4], Zhenyu Li[3]* and Cheng Ma[1,2,6]*

[1]Hefei National Research Center for Physical Sciences at the Microscale, University of Science and Technology of China, Hefei, Anhui 230026, China.

[2]CAS Key Laboratory of Materials for Energy Conversion, Department of Materials Science and Engineering, University of Science and Technology of China, Hefei, Anhui 230026, China.

[3]Key Laboratory of Precision and Intelligent Chemistry, University of Science and Technology of China, Hefei, Anhui 230026, China.

[4]School of Materials Science and Engineering, State Key Laboratory of New Ceramics and Fine Processing, Tsinghua University, Beijing 100084, China.

[5]Foshan (Southern China) Institute for New Materials, Foshan, Guangdong 528200, China.

[6]National Synchrotron Radiation Laboratory, Hefei, Anhui 230026, China.

[†]These authors contributed equally to this work.

*Corresponding author: mach16@ustc.edu.cn; zyli@ustc.edu.cn

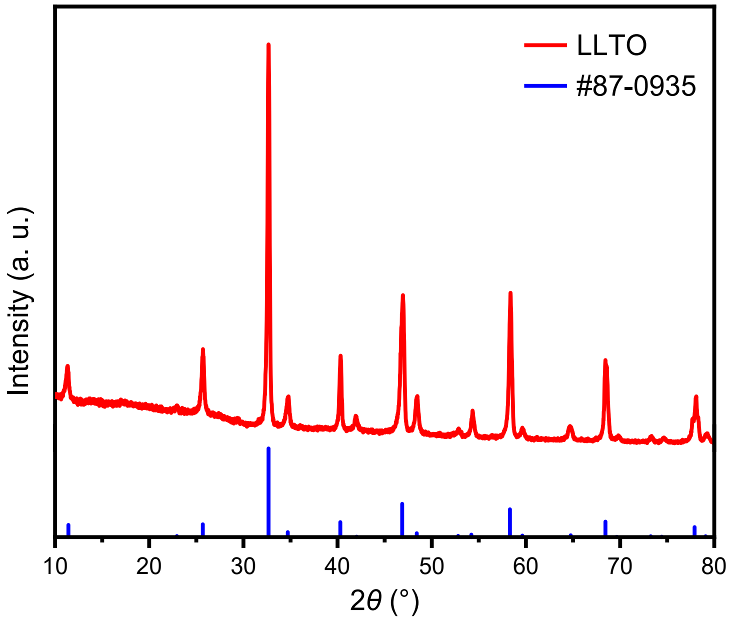

Supplementary Fig. 1 | X-ray diffraction pattern of LLTO. The diffraction pattern agrees well with that of the tetragonal LLTO (PDF#87-0935).

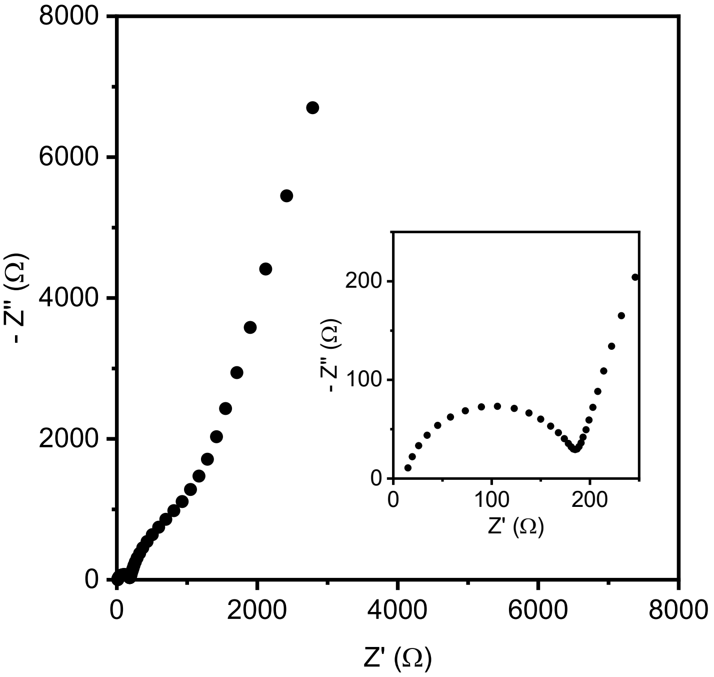

Supplementary Fig. 2 | Nyquist plot of LLTO at 25 °C. The high-frequency, smaller semicircle corresponds to the bulk resistance, and the low-frequency, larger semicircle corresponds to the grain-boundary resistance. The bulk, grain-boundary, and total conductivities are 8.95×10^{-4}, 5.59×10^{-5}, and 5.26×10^{-5} S cm^{-1}, respectively.

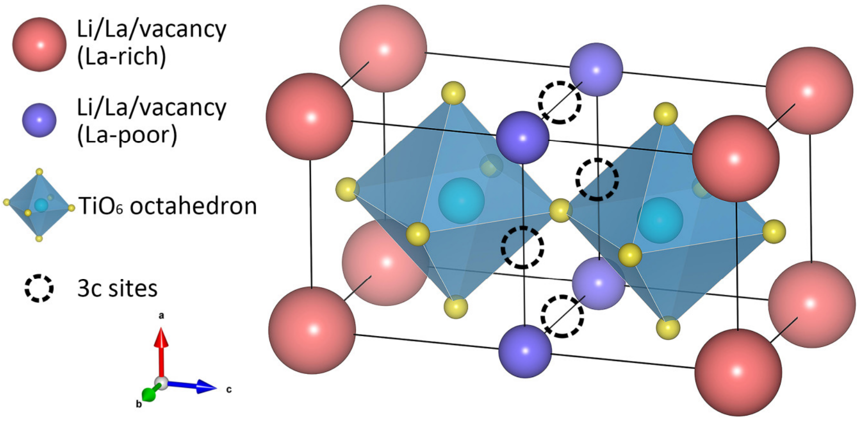

Supplementary Fig. 3 | Location of the 3c interstitial sites. The 3c interstitial sites are located between two neighboring La-poor sites, as indicated by the dashed circles.

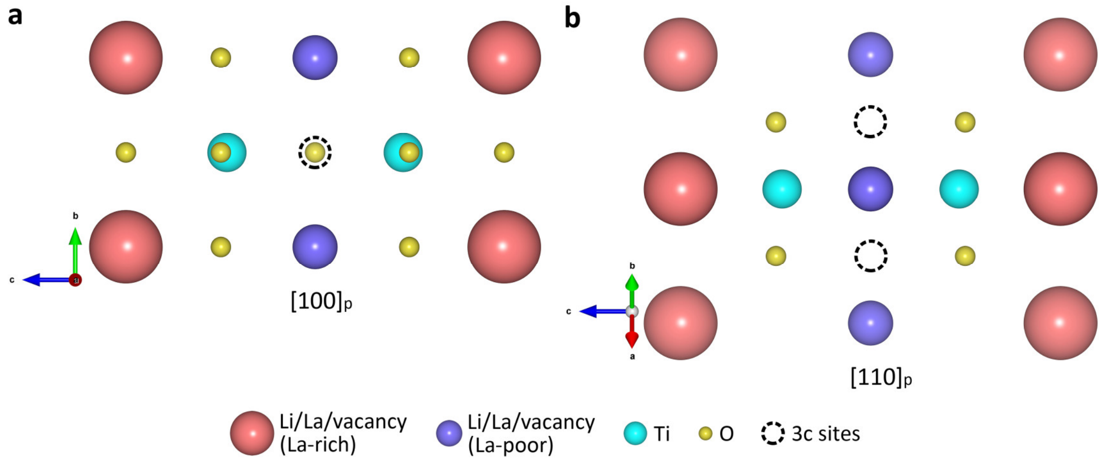

Supplementary Fig. 4 | Appropriate orientation to visualize 3c interstitials. a–b The unit cells of LLTO viewed along $[100]_p$ (**a**) and $[110]_p$ (**b**).

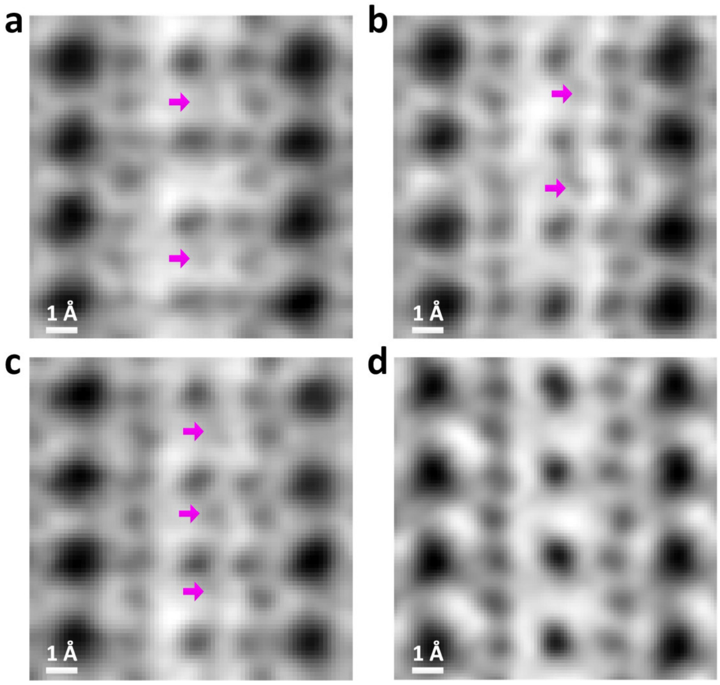

Supplementary Fig. 5 | Li locations in the SCLs and the bulk. **a**–**c** High-magnification ABF-STEM images of regions I (**a**), II (**b**), and III (**c**) in Fig. 3a of the main text. False colors are not applied for these images. **d** ABF-STEM image of the bulk along the same orientation as **a**–**c**, i.e., <110>$_p$. The magnification is the same for all the four images presented here.

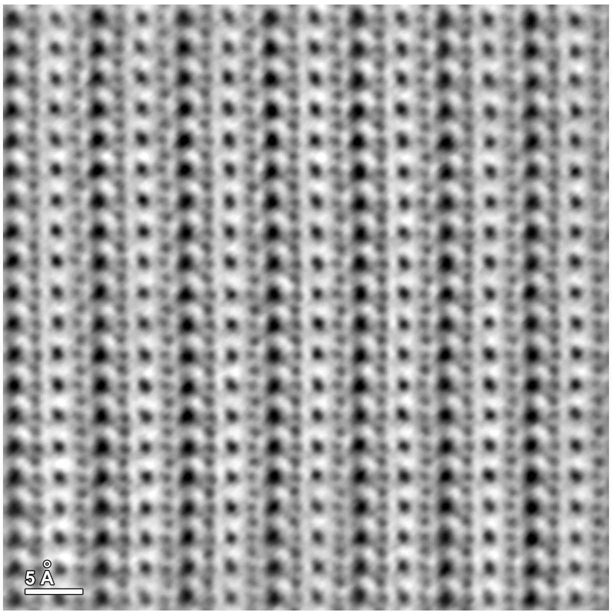

Supplementary Fig. 6 | ABF image of the bulk with relatively large field of view. The viewing direction is <110>$_p$. None of the unit cells in this image exhibits Li near the 3c interstitial site.

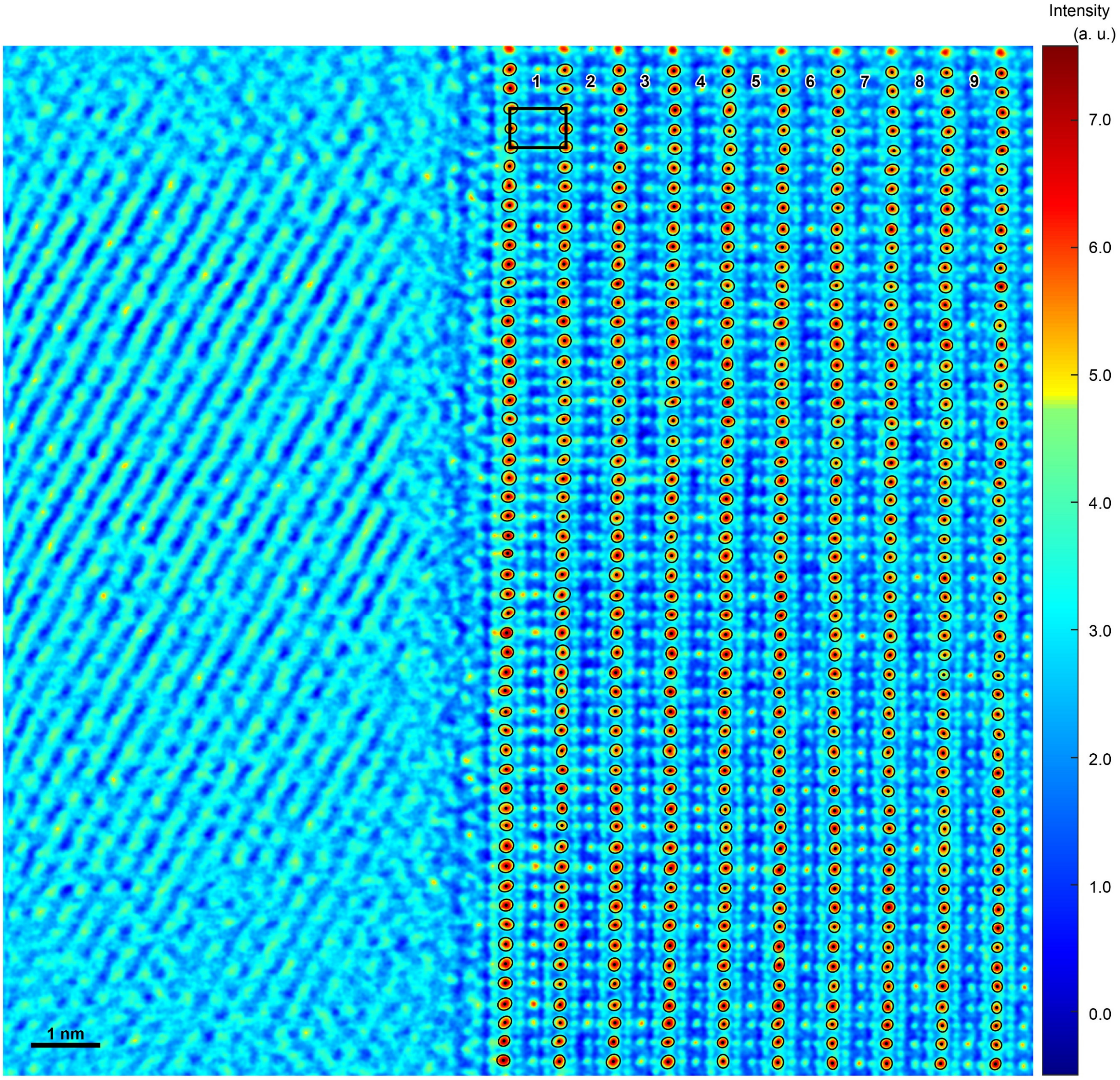

Supplementary Fig. 7 | Identifying the unit-cell volumes through the multiple-ellipse fitting. The fitting was conducted on the image in Fig. 3a of the main text using the CalAtom software. The black rectangle delineates one of the unit cells. Each column of unit cells is assigned with a number for further discussion.

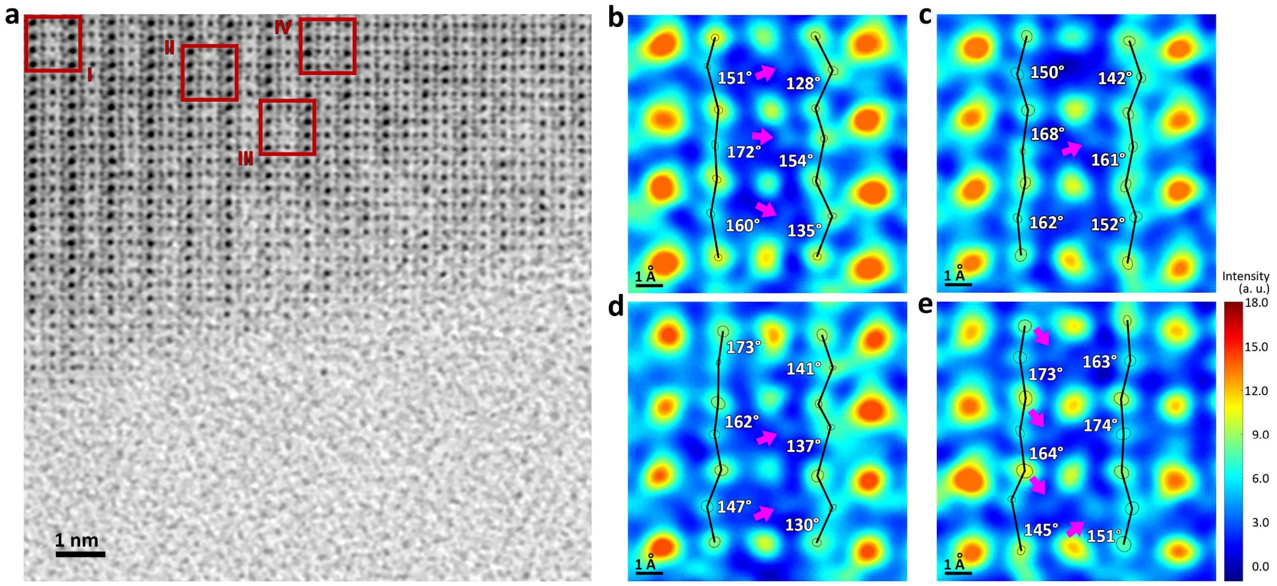

Supplementary Fig. 8 | Atomic configuration of the SCL near a grain boundary. a ABF-STEM image of the vicinity of a grain-boundary core. The upper grain was observed along $\langle 110 \rangle_p$. **b**–**e** Enlarged ABF-STEM images of regions I (**b**), II (**c**), III (**d**) and IV (**e**) in **a**. The magnifications of these enlarged images are the same. For clarity, the images are presented in false colors. The Ti-O bonds are represented by the black lines. The interstitial Li are arrowed in red. The Ti-O-Ti angels were determined by CalAtom.

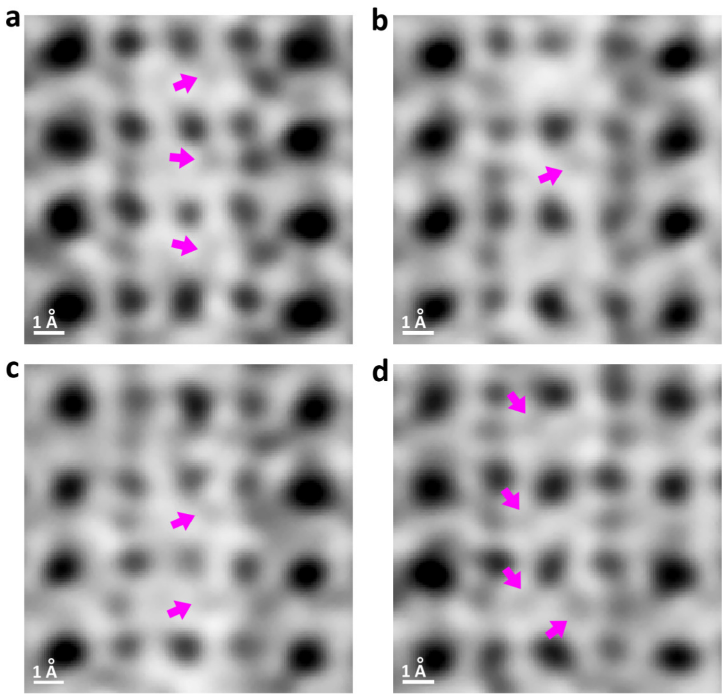

Supplementary Fig. 9 | Li locations in the SCL near a grain boundary. a–d High-magnification ABF-STEM images of regions I (**a**), II (**b**), III (**c**) and IV (**d**) in Supplementary Fig. 8a. For all these images, the false colors are not applied, and the magnifications are the same. The interstitial Li are arrowed in red.

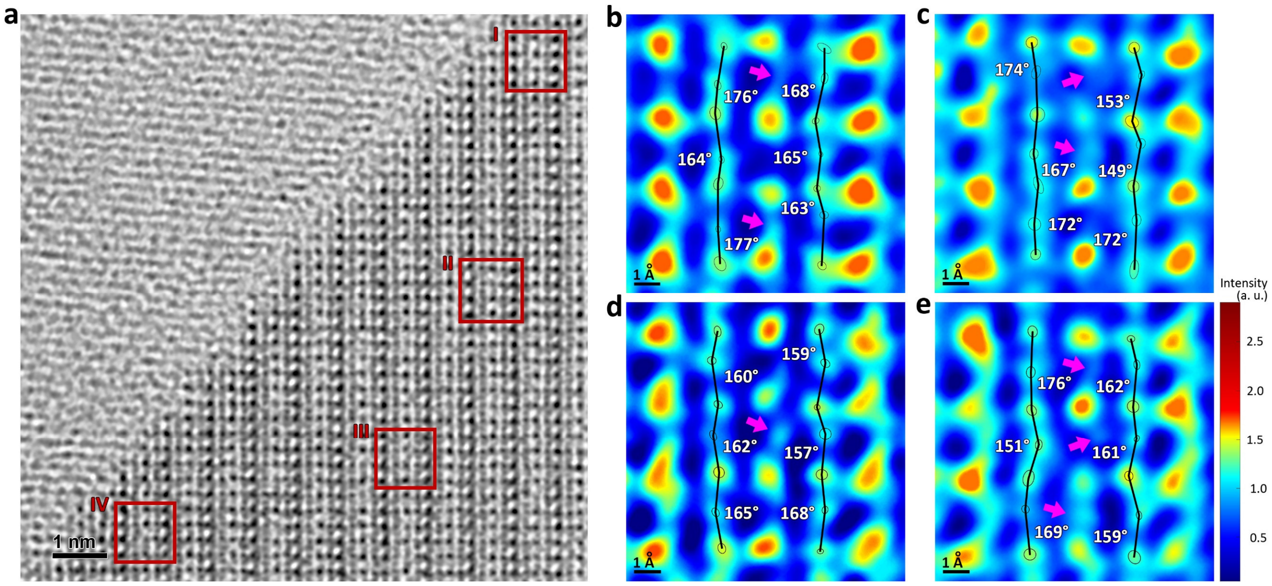

Supplementary Fig. 10 | Atomic configuration of the SCL near a grain boundary. a ABF-STEM image of the vicinity of a grain-boundary core. The grain at the lower right was observed along $<110>_p$. **b**–**e** Enlarged ABF-STEM images of regions I (**b**), II (**c**), III (**d**) and IV (**e**) in **a**. The magnifications of these enlarged images are the same. For clarity, the images are presented in false colors. The Ti-O bonds are represented by the black lines. The interstitial Li are arrowed in red. The Ti-O-Ti angels were determined by CalAtom.

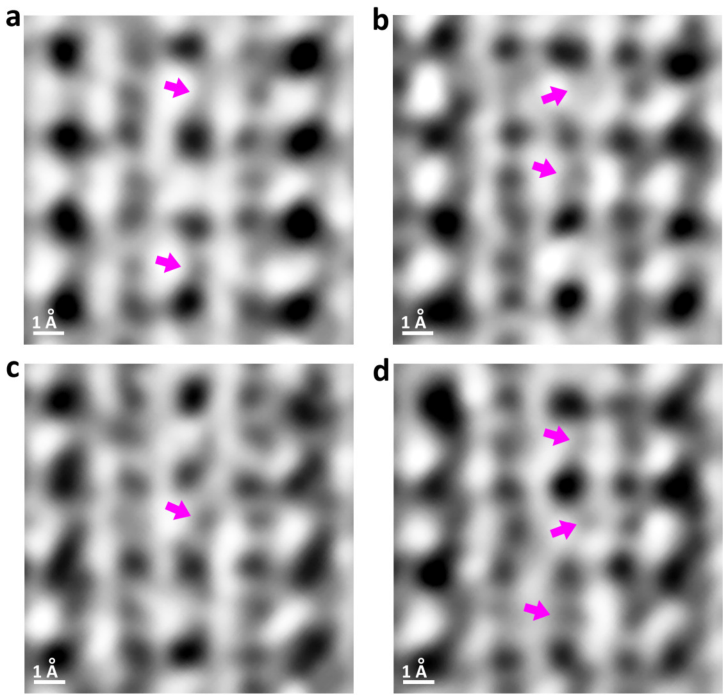

Supplementary Fig. 11 | Li locations in the SCL near a grain boundary. a–d High-magnification ABF-STEM images of regions I (**a**), II (**b**), III (**c**) and IV (**d**) in Supplementary Fig. 10a. For all these images, the false colors are not applied, and the magnifications are the same. The interstitial Li are arrowed in red.

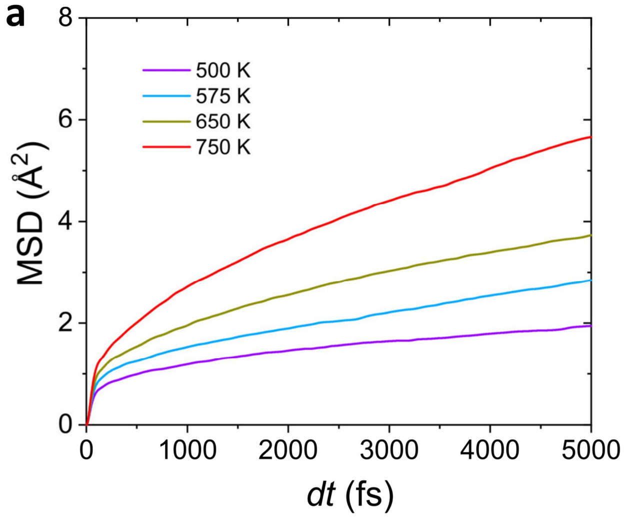

Supplementary Fig. 12 | Computed mean square displacements (MSDs) of Li-ion diffusion. **a**–**b** MSDs of $Li_{0.33}La_{0.56}TiO_3$ (**a**) and those of $Li_{0.66}La_{0.56}TiO_3$ (**b**) at 500, 575, 650, and 750 K. The MSD data beyond 2000 fs were used to calculate the diffusion coefficients.

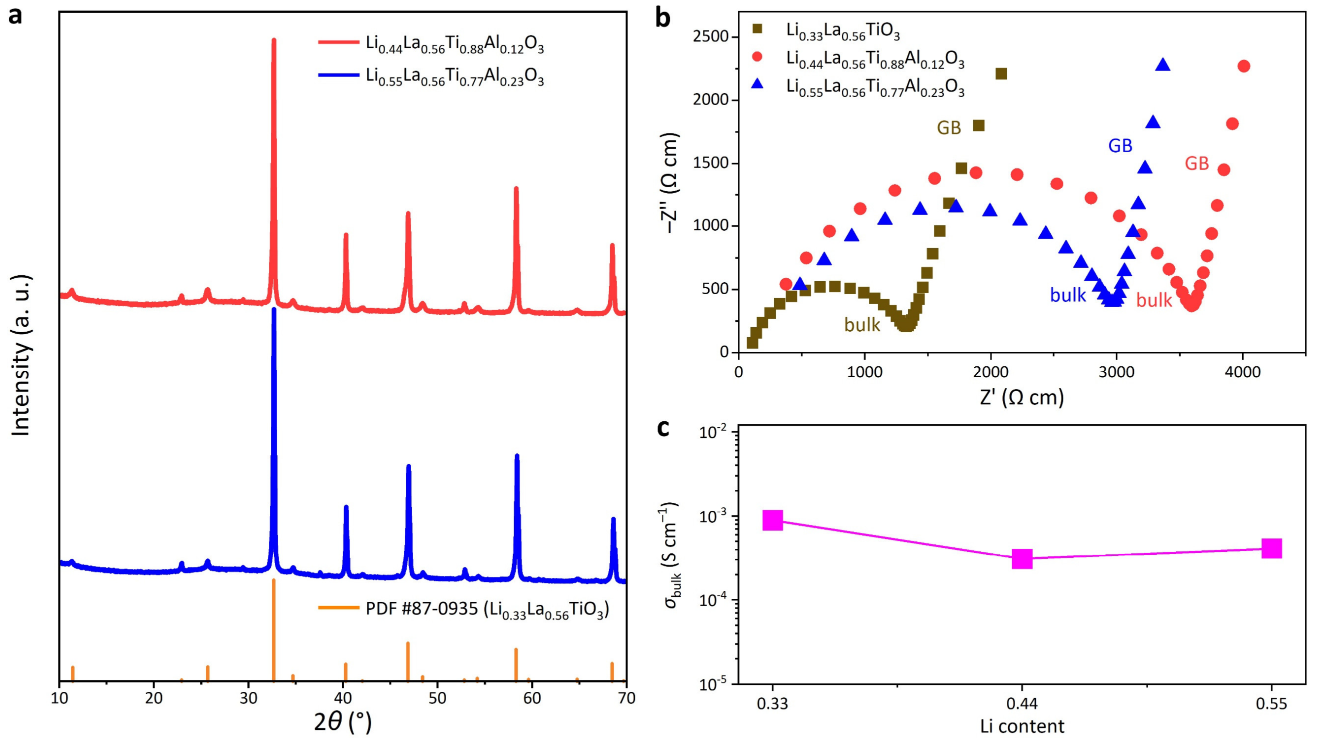

Supplementary Fig. 13 | Experimental verification of the computed diffusion behavior. **a** XRD patterns of $Li_{0.44}La_{0.56}Ti_{0.88}Al_{0.12}O_3$ and $Li_{0.55}La_{0.56}Ti_{0.77}Al_{0.23}O_3$. **b** Nyquist plots of $Li_{0.33}La_{0.56}TiO_3$, $Li_{0.44}La_{0.56}Ti_{0.88}Al_{0.12}O_3$, and $Li_{0.55}La_{0.56}Ti_{0.77}Al_{0.23}O_3$ at 25 °C. The grain-boundary (GB) and bulk semicircles are clearly distinguishable for all the three materials, but the grain-boundary semicircles are not shown completely to allow for a more straightforward comparison of the bulk ones. **c**, Variation of the bulk ionic conductivity (σ_{bulk}) at 25 °C with the Li content in the three compositions shown in **b**.

Supplementary Table 1. Average volume of the unit cells without interstitial Li in each column of unit cells in Supplementary Fig. 7.

Column	#1	#2	#3	#4	#5	#6	#7	#8	#9
Number of unit cells without interstitial Li	19	7	12	9	10	6	7	7	5
Average volume of such unit cells ($Å^3$)	127.0	127.0	123.6	128.1	127.3	125.7	125.8	126.0	127.5
